# Digital livestock systems and probiotic mixtures can improve the growth performance of swine by enhancing immune function, cecal bacteria, short-chain fatty acid, and nutrient digestibility

**DOI:** 10.3389/fvets.2023.1126064

**Published:** 2023-03-24

**Authors:** Sang-O Park, Kyung-Hoon Seo

**Affiliations:** ^1^College of Animal Life Science, Kangwon National University, Chuncheon, Republic of Korea; ^2^Hooin Ecobio Institute, Hongseong-gun, Chungcheongnam-do, Republic of Korea

**Keywords:** immune marker, growth performance, digital livestock system, probiotics, swine

## Abstract

In response to climate change, the use of digital livestock systems and probiotic mixtures as technological strategies to improve animal health and production is driving new innovations in the farm animal industry. However, there is little information available regarding the effects of digital livestock systems and probiotic mixtures (consisting of *Bacillus subtillus, Streptomyces galilaeus*, and *Sphingobacteriaceae*) on the growth performance of the growth-finishing swine. Thus, the objective of this study was to investigate the effects of digital livestock systems and probiotic mixtures on the immune function, cecal bacteria, short-chain fatty acids, nutrient digestibility, and growth performance of growth-finishing swine. A total of 64 crossbred male swine (Duroc × Landrace × Yorkshire, average body weight: 60.17 ± 1.25 kg) were randomly assigned to four treatment groups: CON (control group with a conventional livestock system without a probiotic mixture), CON0.4 (a conventional livestock system with a 0.4% probiotic mixture), DLSC (a digital livestock system without a probiotic mixture), and DLS0.4 (a digital livestock system with a 0.4% probiotic mixture). The swine were reared under standard environmental conditions until their average body weight reached 110 kg. The results indicated that the growth performance of the swine improved with an increase in nutrient digestibility and immune function *via* modulation of blood immune markers in the group with a digital livestock system compared to the CON group, although the growth performance of the swine was similar between the DLSC and CON0.4 groups. Moreover, the application of the digital livestock system and the probiotic mixture maintained higher levels of Lactobacillus and balanced short-chain fatty acid profiles compared to the CON group. These results suggest that a digital livestock system and a probiotic mixture can improve the growth performance of swine by enhancing their nutrient digestibility, improving their immune function, and maintaining balanced cecal bacteria and short-chain fatty acids. Therefore, this study provides insights into the application of digital livestock systems and probiotic mixtures as a climate change response strategy to improve swine production.

## Introduction

Climate change-induced global warming can negatively impact animal behavior and welfare through heat stress and greenhouse gas (GHG) emissions, which can ultimately result in economic animal crises and food supply problems. Thus, preemptive responses are required. A digital livestock system has been developed to resolve the farming animal crisis due to climate change and to maintain sustainable livestock production (1, 2). The development of a digital livestock system for precision livestock farming (PLF) has led to the implementation of remote monitoring systems using cameras, microphones, and accelerometer sensors. Rapid progress has been made to improve animal welfare and production, and various systems managed by high-tech equipment have been introduced to livestock farming (1–3). Digital livestock systems, including feeding, weighing, and sorting systems, wearable sensors, and climate control systems, have been implemented in swine farming in an effort to improve swine production, animal behavior and welfare, sound detection, and 3D camera application information (4, 5).

The FAO has predicted that the world's population will reach 9.2 billion by 2050, resulting in a 50–60% increase in food consumption compared to current levels. Global animal food production will increase by 2/3 from 334 million tons in 2015 to 498 million tons in 2050 (6, 7). By 2050, the consumption of meat, eggs, and dairy products is predicted to increase by 73, 64, and 58%, respectively, in comparison with the present situation. Thus, sustainable livestock production is necessary to secure food supplies. The use of precision livestock farming (PLF) as a digital livestock system is being increasingly adopted to improve the swine industry. This climate-smart livestock system has recently been developed in response to the rapid increase in pork meat consumption (8, 9).

In the digital era, an intelligent PLF and ICT (information and communication technology)-based climate-smart livestock system (hereinafter referred to as a digital livestock system) is being commercialized as future megatrend strategies using big data converged with the 4th industrial revolution's ICT, Internet of Things (IoT), wireless biosensors, mobile phones, artificial intelligence (AI), automatic systems, and drone technology (1, 2, 10–12). A digital livestock system is a new technology developed for the animal livestock industry that can improve humans' and animals' lives through ICT convergence, where the demand for information across the supply and food chains is the source of power for the development and expansion of digital agriculture (13–15). A digital livestock system, such as PLF, is a groundbreaking innovation that can play a significant role in addressing climate change issues and improving feeding management, ventilation, and animal welfare by enhancing the quality of livestock production and animal food. It allows for young livestock populations to be reared in rural communities, thus promoting sustainable livestock farming. It can control, adjust, and monitor the ventilation, heating, and cooling of livestock farms through a central PC. It can also remotely control feeding management using a smartphone or tablet PC. Error messages are sent *via* SMS or email. The system is integrated with an air cleaner (14–17). In particular, it can remove specks of dust, ammonia, and odors that are generated from livestock farms during the summer to provide a comfortable climate for livestock. In addition, it can improve livestock production while reducing the environmental impact of the livestock industry, saving energy, and improving animal welfare. The use of a digital livestock system as a technological strategy to improve animal health and production can lead to new innovations in the economic animal industry. It is known to improve economic animals' welfare and growth performance by automating feeding and environmental management systems. However, little is known about its effect on the growth performance of growth-finishing swine (1, 2, 18–20).

Probiotics are living organisms that are part of the normal intestinal microflora of animals. Maintaining optimal levels of intestinal bacteria in swine through the use of probiotics can improve animal production and boost immune function (21–23). Previous studies have reported that a probiotic mixture (*Bacillus subtillus, Streptomyces galilaeus*, and *Sphingobacteriaceae*) can improve growth performance and egg and chicken meat quality by enhancing caecal microbial balance and immune function. The probiotic mixture can also reduce the odor in poultry farms for laying hens and broilers exposed to heat stress (22, 23). However, little is known about the effect of using digital livestock systems and probiotic mixtures on the growth performance of growth-finishing swine (1, 2, 16, 18–20).

This study was conducted to compare the effects of a conventional livestock system, a digital livestock system, and both livestock systems with a probiotic mixture on growth performance, immune function, cecal bacteria, cecal short-chain fatty acid, and nutrient digestibility in growth-finishing swine.

## Materials and methods

### Ethical approval

This study was conducted according to the guidelines for scientific and ethical procedures provided in the European Experimental Animal Handling License (SCT-w94058) and European Union Council Directive 2008/120/EC. It was approved by the Institutional Animal Care and Use Committee of Kangwon National University, Republic of Korea (No. KNU-20197).

### Animals and experimental design

The animal experiment was conducted in swine research farms of the Hooin Ecobio Institute (Hongseong, Chungcheongnamdo), Republic of Korea. A total of 64 crossbred male swine (Duroc × Landrace × Yorkshire; average body weight: 60.17 ± 1.25 kg) were reared until their body weights reached ~110 kg. Regarding the blocking factor for the experiment, the livestock system and probiotic mixtures were treatment variables. The swine were assigned to one of the following four groups (16 replicate pens per group) based on a randomized complete block design: CON (control group with a conventional livestock system without a probiotic mixture), CON0.4 (a conventional livestock system with a 0.4% probiotic mixture), DLSC (digital livestock system without a probiotic mixture), and DLS0.4 (a digital livestock system with a 0.4% probiotic mixture). Mixed probiotics were prepared by mixing 2% of *Bacillus subtillus, Streptomyces galilaeus*, and *Sphingobacteriaceae* (isolated from earthworm cast) with 98% of the earthworm cast. These products were provided by HooinEcobio Co., Ltd. (Korean Patent No. 0092670). Each strain contained at least 3.5 × 10^8^ colony-forming units (CFU/g of product) (22, 23). The level of probiotic mixture added was determined based on preliminary results, demonstrating plateau levels without further increasing growth performance under standard environmental conditions for swine research farms.

### Digital livestock system

The digital livestock system used in this study incorporated ICT convergence, big data, and mobile phones. It was equipped with various features such as automatic feeding and drinking dispensers, radio frequency identification, temperature and humidity sensors, an odor removal system, cooling pads, an air conditioner, livestock farm monitoring through CCTV, feed bins, and a mobile imaging system for live weight measurement (1, 2, 16).

### Diets and feeding management

This study used experimental basal diets formulated based on the nutritional requirements for growth-finishing swine recommended by the NRC (24). The probiotic mixture was added to the yellow corn grain. Ingredients and chemical compositions of the basal diet are shown in Table 1. The swine were reared under the same standard environmental conditions (a breeding area of 0.45–0.8 m^2^ per animal, a temperature of 25°C, and a relative humidity of 50–60%) for both livestock systems. Through an installed ventilation controller, minimum ventilation and maximum ventilation were adjusted to 20–30% and 60–70%, respectively, to minimize changes in the internal environment in line with changes in the external environment. All animals were provided free access to water and feed. After feeding basal diets for 45 days, a total of 24 animals (six replicates per group, four groups) were moved to metabolic cages to measure nutrient digestibility. The remaining 40 animals (10 replicates per group, four groups) were kept to investigate growth performance.

**Table 1:** Formula and chemical composition of basal diet (as-fed basis).

**Items**	**%**
Yellow corn grain	47.58
Soybean oil meal	14.20
Lupine seed	11.00
Wheat grain	10.00
Rapeseed	3.00
Distiller's dried grains soluble	3.00
Beef tallow	5.00
Cane molasses	3.00
Limestone	0.94
Dicalcium phosphate	1.17
Common salt	0.30
Yeast culture	0.18
Mineral premix^a^	0.15
Vitamin premix^b^	0.10
Threonine 96%	0.05
L-lysine hydrochloride 98%	0.33
**Chemical composition, %**	
Digestible energy MJ/kg^c^	12.60
Crude protein	17.00
Lysine	0.97
Cystine	0.27
Methionine	0.25
Calcium	0.77
Available phosphorous	0.38

a Mineral premxc contained per kg of diets Cu 200 mg, Fe 80 mg, Zn 180 mg, Mn 13 mg, I 0.4 mg, Co 0.15 mg, Se 0.35 mg.

b Vitamin premix contained per kg of diet Vit. A 20,000 IU, Vit. D3 4,000 IU, Vit. E 75 IU, Vit. K3 12 mg, Vit. B24 mg, Vit. B6 1 mg, Vit. B12 60 μg, Pantothenic acid 50 mg, Niacin 120 mg, Biotin 0.08 mg.

c Digestible energy (calculated value by NRC, 2012).

### Growth performance and spleen weight

The growth performance was measured based on average daily weight gain (ADG), average daily feed intake (ADF), and the feed conversion ratio (FCR) (weight gain/feed intake) at the beginning and end of animal experiments, respectively. After finishing the animal feeding experiment, the swine were euthanized using electrical stunning and exsanguination. The weights of their spleens (immune organ; as % BW, the relative weight to body weight) were then measured.

### Nutrient digestibility

The digestibility of nutrients was measured for six replicates per group in four groups. The animals were moved into metabolic cages for fecal collection. They were fed different diets mixed with 0.30% chromic oxide as an indigestible marker to measure their nutrient digestibility. The animals had an adaptation period of 1 week. Total feces from each metabolic cage were collected for 3 days. After collection, fecal samples were oven-dried at 70°C and milled through a 40-mesh sieve prior to chemical analyses. Nutrient compositions and chromic oxide in diets and fecal samples were analyzed following the AOAC testing methods (25). Nutrient digestibility was calculated as a percentage: (the amount of marker in the diet/amount of marker in the feces) × (amount of nutrient in the feces/amount of nutrient in the diet) (26).

### Immune markers

To evaluate the immune markers of animals at the end of the experiment, we collected 10 mL of blood from the jugular vein of each animal and placed it into a clot activator vacuum tube (Becton Dickinson Vacutainer Systems, Franklin Lakes, NJ, USA). Immunoglobulin G (IgG) concentration in serum was measured using a swine IgG ELISA Kit (E101-104, Bethyl Laboratories, USA). The serum concentration of cortisol, a stress hormone, was quantified using a swine cortisol ELISA Kit (EKC31445-BM, BioCat GmbH, Germany) according to the manufacturer's protocol. Three blood smears per animal were taken immediately after blood collection, and the slides were stained with the May-Grunwald-Giemsa stain after air drying. Heterophils (H) and lymphocytes (L) were differentiated through light microscopy. After 100 leukocytes per slide and 200 cells per animal were counted, the H/L ratio was then calculated (27).

### Cecal bacteria counts

*Lactobacillus, E. coli*, coliform bacteria, and total aerobic bacteria from cecal contents were cultured on MRS agar (Difco), violet red bile agar with MUG (Difco), and nutrient agar (Difco), respectively. After aerobic culturing at 37°C for 24 h, the count of colonies for each strain was determined using a colony counter. Data were presented as log10 colony forming units (CFU) per gram of cecal content (28).

### Cecal short-chain fatty acids

Cecum samples were collected from each swine to analyze short-chain fatty acids (SCFAs) and bacteria counts. The cecum was quickly frozen in liquid nitrogen and then stored at −80°C for further analysis. Cecal SCFAs were measured using a gas chromatographic system (model GC-15A, Shimadzu Corp., Kyoto, Japan) equipped with a glass column filled with 10% SP-1000/1% H_3_PO_4_ (180 cm × 4 mm; Supelco, Inc., Bellefonte, PA, US) and attached to a flame ionization detector. After adding 25% H_3_PO_4_ solution to the supernatant, the mixture was homogenized, maintained on ice for more than 30 min, and then centrifuged at 3,000 rpm for 10 min at 4°C (29).

### Statistical analysis

All obtained data were checked for normal distribution using the Shapiro–Wilk normality test prior to analysis. They were analyzed using the generalized linear model (GLM) procedure of SAS (SAS 9.4 Institute Inc., Cary, NC, USA) as a randomized complete block design with a repeat pen serving as the experimental unit. The effects of the digital livestock system, conventional livestock system, and probiotic mixtures were analyzed with an individual animal taken as an experimental unit. A linear effect analysis was performed using polynomial orthogonal contrasts. Differences among treatments were separated using Tukey's multiple-range test. Probability values < 0.05 were considered significant (*P* < 0.05).

## Results

### Growth performance

The results revealed that the applied digital livestock system greatly improved the growth performance of swine compared to the conventional livestock system without a probiotic mixture. However, although the DLSC and CON0.4 groups showed the same results (Table 2), ADG was significantly increased in the order of the DLS0.4, DLSC = CON0.4, and CON groups (*P* < 0.05). ADF was significantly higher in the order of the CON, CON0.4 = DLSC, and DLS0.4 groups (*P* < 0.05). FCR was significantly lower in the order of the DLS0.4, DLSC = CON0.4, and CON groups due to increased ADF (*P* < 0.05).

**Table 2:** Growth performance of growth-finishing swine reared under digital livestock system (60–110 kg body weight).

**Items**	**Groups** ^**1**^					
	**CON**	**CON0.4**	**DLSC**	**DLS0.4**	**SEM** ^3^	***P*** **-value**
ADG^2^, g/d	784^c^	807^b^	805^b^	824^a^	27.05	0.010
ADF, g/d	2,406^a^	2,229^b^	2,240^b^	2,158^c^	89.17	0.019
FCR	3.07^a^	2.76^b^	2.78^b^	2.62^c^	0.098	0.010

1 CON (control group with a conventional livestock system without a probiotic mixture), CON0.4 (conventional livestock system with a 0.4% probiotic mixture), DLSC (digital livestock system without a probiotic mixture), and DLS0.4 (digital livestock system with a 0.4% probiotic mixture).

2 ADG: average daily weight gain, ADF: average daily feed intake, FCR: the feed conversion ratio, ADF:ADG.

3 Standard error of mean values, *n* = 10.

a,b,c,d Mean values with different superscripts are significantly different at *P* < 0.05.

### Nutrient digestibility

The use of the digital livestock system significantly increased nutrient digestibility compared to the conventional livestock system without a probiotic mixture, although the DLSC and CON0.4 groups showed a similar trend (Table 3). Regarding the digestibility of dry matter, crude protein was significantly increased in the order of the DLS0.4, DLSC = CON0.4, and CON groups. The ether extract was higher in the order of the DLS0.4, DLSC, CON0.4, and CON groups, and crude ash was significantly increased in the order of the DLS0.4, DLSC = CON0.4, and CON groups (*P* < 0.05).

**Table 3:** Nutrient digestibility of growth-finishing swine reared under digital livestock system.

**Items**	**Groups** ^**1**^					
	**CON**	**CON0.4**	**DLSC**	**DLS0.4**	**SEM** ^2^	***P*** **-value**
Dry matter, %	89.22^c^	91.37^b^	92.12^b^	93.04^a^	3.015	0.021
Crude protein, %	88.21^c^	90.79^b^	91.04^b^	92.85^a^	3.507	0.027
Ether extract, %	77.71^d^	80.16^c^	83.45^b^	87.32^a^	2.365	0.030
Crude ash, %	76.52^b^	77.56^b^	78.01^b^	80.20^a^	2.880	0.017

1 CON (control group with a conventional livestock system without a probiotic mixture), CON0.4 (conventional livestock system with a 0.4% probiotic mixture), DLSC (digital livestock system without a probiotic mixture), and DLS0.4 (digital livestock system with a 0.4% probiotic mixture).

2 Standard error of mean values, *n* = 6.

a,b,c,d Mean values with different superscripts are significantly different at *P* < 0.05.

### Immune markers

The digital livestock system greatly improved the immune function of animals by modulating serum immune markers compared to the conventional livestock system without a probiotic mixture, although the DLSC and CON0.4 groups showed a similar trend (Table 4). Serum immune markers, IgG, cortisol, lymphocytes (L), and the H/L ratio were significantly increased in the order of the CON, CON0.4 = DLSC, and DLS0.4 groups (*P* < 0.05). Heterophil (H) was significantly higher in order of the CON, DLSC, CON0.4, and DLS0.4 groups (*P* < 0.05). The weights of the spleen (ranging from 0.11–0.13 % BW) as an immune organ did not differ between the groups.

**Table 4:** Blood biomarkers and spleen weight in growth-finishing swine reared under.

**Items**	**Groups** ^**1**^					
	**CON**	**CON0.4**	**DLSC**	**DLS0.4**	**SEM** ^2^	***P*** **-value**
IgG, μg/mL	250.1^a^	208.7^b^	211.3^b^	156.30^c^	8.330	0.027
Cortisol, μg/dL	7.21^a^	5.17^b^	6.01^b^	4.28^c^	0.242	0.010
Heterophils (H), %	127.8^a^	88.17^c^	93.78^b^	65.72^d^	0.892	0.010
Lymphocytes (L), %	27.85^a^	25.70^b^	26.03^b^	21.72^c^	3.102	0.010
H/L ratio	4.59^a^	3.43^b^	3.60^b^	3.02^c^	0.009	0.001
Spleen, % BW	0.11	0.12	0.13	0.12	0.003	0.001

1 CON (control group with a conventional livestock system without a probiotic mixture), CON0.4 (conventional livestock system with a 0.4% probiotic mixture), DLSC (digital livestock system without a probiotic mixture), and DLS0.4 (digital livestock system with a 0.4% probiotic mixture).

2 Standard error of mean values, *n* = 10.

a,b,c Mean values with different superscripts are significantly different at *P* < 0.05.

### Short-chain fatty acid

The digital livestock system maintained balanced short-chain fatty acid (SCFA) profiles compared to the conventional livestock system without a probiotic mixture, although the DLSC and CON0.4 groups showed a similar trend (Table 5). In comparison to the SCFAs of the CON and DLSC groups, the SCFAs of the DLS0.4 and CON0.4 groups exhibited a significant increase in acetic acid and propionic acid but not in butyric acid, isobutyric acid, valeric acid, or isovaleric acid. Cecal total SCFA was significantly increased in the order of the DLS0.4, CON0.4, and DLSC = CON groups (*P* < 0.05). Acetic acid and propionic acid were significantly increased in the order of the DLS0.4 = CON0.4, DLSC, and CON groups (*P* < 0.05). Butyric acid was significantly higher in the CON group than in the other groups (*P* < 0.05). Isobutyric acid was significantly increased in the order of the CON, DLSC, and DLS0.4 = CON0.4 groups (*P* < 0.05). Valeric acid and isovaleric acid were significantly higher in the CON group than in other groups (*P* < 0.05).

**Table 5:** Cecal short-chain fatty acid in growth-finishing swine reared under a digital livestock system (SCFA, μmol/g of cecal content).

**Items**	**Groups** ^**1**^					
	**CON**	**CON0.4**	**DLSC**	**DLS0.4**	**SEM** ^2^	***P*** **-value**
Acetic acid	80.10^c^	101.2^a^	98.04^b^	103.5^a^	3.160	0.010
Propionic acid	16.72^c^	25.17^a^	20.32^b^	26.33^a^	0.875	0.022
Butyric acid	27.18^a^	15.70^b^	16.33^b^	15.78^b^	0.622	0.013
Isobutyric acid	20.08^a^	12.41^c^	14.78^b^	13.07^c^	0.502	0.016
Valeric acid	7.75^a^	6.14^b^	6.20^b^	5.52^b^	0.243	0.010
Isovaleric acid	5.84^a^	3.06^b^	3.55^b^	2.85^b^	0.153	0.008
Total SCFA	157.7^c^	163.8^b^	159^c^	167.1^a^	5.366	0.035

1 CON (control group with a conventional livestock system without a probiotic mixture), CON0.4 (conventional livestock system with a 0.4% probiotic mixture), DLSC (digital livestock system without a probiotic mixture), and DLS0.4 (digital livestock system with a 0.4% probiotic mixture).

2 Standard error of mean values, *n* = 10.

a,b,c Mean values with different superscripts are significantly different at *P* < 0.05.

### Cecal bacteria

The digital livestock system increased *Lactobacillus* but decreased *E. coli* counts compared to the conventional livestock system without a probiotic mixture (Table 6). Cecal *Lactobacillus* counts were significantly lower in the CON group than in other groups (*P* < 0.05), but they showed no significant differences among the DLSC, DLSC0.4, and CON0.4 groups. *E. coli* counts were significantly increased in the order of the CON, DLSC, CON0.4, and DLS0.4 groups (*P* < 0.05). Coliform bacteria counts were significantly increased in the order of the CON, DLSC, CON0.4, and DLS0.4 groups (*P* < 0.05), although they were not significantly different between the CON0.4 and DLS0.4 groups. Total aerobic bacteria counts were significantly increased in the order of the CON, DLSC = CON0.4, and DLS0.4 groups (*P* < 0.05), although they were not significantly different between the DLSC and CON0.4 groups (*P* < 0.05).

**Table 6:** Cecal bacteria counts in growth-finishing swine reared under a digital livestock system (log cfu/g of fresh cecal content).

**Items**	**Groups** ^**1**^					
	**CON**	**CON0.4**	**DLSC**	**DLS0.4**	**SEM** ^2^	***P*** **-value**
*Lactobacillus*	6.71^b^	8.52^a^	8.13^a^	8.15^a^	0.260	0.010
*E. coli*	8.12^a^	6.64^c^	7.01^b^	5.07^d^	0.254	0.010
Coliform bacteria	7.72^a^	5.01^c^	6.32^b^	4.83^c^	0.198	0.008
Total aerobic bacteria	7.55^a^	6.17^b^	6.40^b^	5.08^c^	0.201	0.010

1 CON (control group with a conventional livestock system without a probiotic mixture), CON0.4 (conventional livestock system with a 0.4% probiotic mixture), DLSC (digital livestock system without a probiotic mixture), and DLS0.4 (digital livestock system with a 0.4% probiotic mixture).

2 Standard error of mean values, *n* = 10.

a,b,c,d Mean values with different superscripts are significantly different at *P* < 0.05.

## Discussion

Our results revealed a new fact: the growth performance of swine could be greatly improved by using a digital livestock system compared to a conventional livestock system without a probiotic mixture. Both the DLSC and CON0.4 groups showed a similar trend. Our results also showed that the digital livestock system can improve the growth performance of swine by improving their welfare, reducing stress, and promoting their overall health through the convenience provided by automatic feeding and environmental management, which streamlines animal care. The digital livestock system can also modulate immune markers, increase nutrient digestibility, and balance cecal bacteria and SCFA. However, it is important to note that butyrate SFCA was significantly reduced in the CON0.4, DLSC, and DLS0.4 groups compared to the CON group. This reduction may have negative implications, as butyrate is a crucial SCFA involved in many physiological pathways. Similarly, IgG was significantly decreased in the CON0.4, DLSC, and DLS0.4 groups, and it may be seen as a negative immune response in these groups (1, 5, 17, 19). The fact that DLS0.4 improved the growth performance of swine could be considered a combined effect of the digital livestock system and the probiotic mixture in SCFA (1, 2, 16) (Table 2). CON0.4 and DLS0.4 groups consumed the same level of probiotics, with DLSC and CON0.4 groups showing the same results, suggesting that swine production could be significantly improved by the digital livestock system with the probiotic mixture. However, few such studies have been reported. The digestibility of dry matter, crude protein, crude fat, and crude fiber were higher in the digital livestock system than in the conventional livestock system without a probiotic mixture, although the DLSC and CON0.4 groups showed similar trends, demonstrating improved growth performance in the DLSC group. The digital livestock system resulted in better nutrient digestibility because animal stress was alleviated due to an automatic environment and feeding management control in the digital swine production system, leading to improved nutritional and metabolic capability (1, 16) (Table 3). Animal stresses occur due to various factors, including environmental conditions. These stresses can degrade an animal's nutritional and metabolic capabilities, resulting in decreased digestibility, reduced feed intake, and impaired growth performance (18, 27–29). This supports the results of this study. The fact that the DLS0.4 group showed the highest nutrient digestibility might be due to the possible interaction between the probiotic mixture and the digital livestock system, which ultimately contributed to the improvement of swine production. The supply of probiotics to swine is known to improve nutrient digestibility and increase animal growth performance and production (22, 30, 31).

In the hematological evaluation of animal stress, leukocyte counts (leukocyte profiles) from blood smears, that is, the H/L ratio as an immunological marker, are important. Various stressors, including animal environment and management, can increase blood IgG, H/L ratio, and cortisol concentration as immune markers, enabling it to cope with stress (27, 29, 32). Although spleen weight was not significantly different between treatment groups, the modulation of immune markers in the DLS0.4 group animals could be due to the combined effect of the probiotic mixture and digital livestock systems. This system can promote immune function by increasing nutrient digestibility through feeding intake stimulation and balancing cecal bacteria with the help of probiotics.

In the field of poultry farming, implementing a digital livestock system has been shown to improve animal growth performance and animal welfare while relieving animal stress by modulating immune markers (1, 16). Based on this result, modulated immune markers in the DLS0.4 group might be due to a combined effect of the probiotic mixture and the digital livestock system, thus improving the growth performance of the swine. The growth performance of animals could be improved by modulating immune markers by DLS compared to those of the CON group, although the spleen weight was similar between treatment groups (32, 33) (Table 4). When animals are exposed to poor welfare conditions, poor feeding management, and environmental stress, their blood IgG, H/L ratio, and cortisol concentration rapidly increase as a response. Therefore, proper maintenance of immune markers is crucial to ensure optimal immune function (16, 22, 32). One of the most important roles of the microorganisms found in probiotics is to stimulate immune function in host animals. Various types of probiotics, including normal microorganisms in the digestive tract, are known to stimulate the immune system in animals (21, 22, 30, 34).

It was confirmed that the digital livestock system could maintain balanced cecal SCFA profiles compared to a conventional livestock system without a probiotic mixture, although similar results were observed between the CON0.4 and DLS0.4 groups (Table 5). Probiotics have been known to increase swine production through competition with other intestinal bacteria for nutrients, production of antibacterial substances, enhancement of immune markers, SCFA production, and nutrient digestibility (22, 30, 31). For this reason, it can be seen that the SCFA results between the CON0.4 and DLS0.4 groups showed similar trends.

The obtained data indicated that cecal total short-chain fatty acids, acetic acid, and propionic acid were increased in the DLS0.4 and CON0.4 groups compared to those in the CON and DLSC groups, whereas butyric acid, isobutyric acid, valeric acid, and isovaleric acid showed a higher tendency in the CON group than in other groups, eventually stimulating immune cell development and maintaining balanced levels of immune markers. The results for SCFA of both probiotic mixture groups presented in Table 5 will ultimately contribute to improved swine production through improved immune function and gut health, a reduction in stress, and an improvement in animal welfare (1, 28, 29, 35) (Table 5). Probiotics are known to improve growth performance and immune function by modulating the level of SCFA in the cecum of animals (1, 36–38). However, the combined effect of the digital livestock system and probiotic mixture has not been reported until now.

Our results showed that the digital livestock system could lead to higher cecal *Lactobacillus* counts compared to the conventional livestock system without a probiotic mixture because the CON0.4, DLSC, and DLS0.4 groups displayed the same results. *Lactobacillus*, a good cecal bacteria, can improve the growth performance of animals and maintain the gut environment by balancing cecal bacteria and promoting the stability of indigenous microbes against animal stressors. The digital livestock system can help maintain balanced cecal bacteria by increasing *Lactobacillus* and reducing *E. coli* in the digestive tract of animals, thereby reducing stress and improving the animal's nutritional and metabolic capabilities (1, 16) (Table 6). *E. coli* is known to form a colony of harmful bacteria in intestinal villous cells, generating toxins that can trigger a swine's digestive tract to secrete intestinal fluids, resulting in diarrhea (16, 21, 31). In an environment where animals are exposed to various stressors, unexpected disorders or illnesses will occur if the stability of the intestinal microbial ecosystem is threatened (1, 21, 29, 31). For this reason, in this study, the growth performance of the swine was improved with the digital livestock system.

## Conclusion

In conclusion, our study demonstrated that the use of a digital livestock system can improve the growth performance of swine compared to both a conventional livestock system without a probiotic mixture and a conventional system with a probiotic mixture. This study's findings provide new insights into the action mechanism through which the digital livestock system and probiotic mixture can improve the growth performance of swine by reducing stress, increasing nutrient digestibility, promoting a balanced cecal bacterial community and SCFA production, and modulating immune function. The implementation of a digital livestock system can improve animal welfare, animal feeding, and environmental management through automation, wireless mobile phones, and sensing application technology that incorporates ICT convergence. Our results also showed that the growth performance of swine in a digital livestock system without a probiotic mixture and a conventional livestock system with a probiotic mixture were the same. This might be due to the digital livestock system's ability to increase nutrient digestibility, modulate immune function, and balance cecal bacteria and short-chain fatty acid profiles. Immune markers such as IgG, cortisol, and the heterophils (H)/lymphocytes (L) were higher in the digital livestock system than in the conventional livestock system without a probiotic mixture, indicating that the digital livestock system could modulate immune function in swine. Moreover, the digital livestock system modulated the balanced cecal SCFA profiles and bacteria counts, such as *Lactobacillus* and *E. co*li, compared to conventional livestock systems without probiotics. These findings suggest that the digital livestock system could play an important role in maintaining the immune function of swine. The study's results provide valuable insights into the application of a digital livestock system and probiotic mixtures in farm animals as a climate change response strategy, especially in the swine industry.

## Data availability statement

The original contributions presented in the study are included in the article/supplementary material, further inquiries can be directed to the corresponding author.

## Ethics statement

The animal study was reviewed and approved by Institutional Animal Care and Use Committee of Kangwon National University, Republic of Korea (No: KNU-20197). Written informed consent was obtained from the owners for the participation of their animals in this study.

## Author contributions

S-OP designed and conducted the experiments, wrote the original draft, and revised the manuscript. K-HS helped with the animal experiments. S-OP and K-HS analyzed the data. All authors have read and approved the final version of the manuscript.
